# Hepatitis C Virus Infection Increases Risk of Developing End-Stage Renal Disease Using Competing Risk Analysis

**DOI:** 10.1371/journal.pone.0100790

**Published:** 2014-06-27

**Authors:** Jia-Jung Lee, Ming-Yen Lin, Jung-San Chang, Chi-Chih Hung, Jer-Ming Chang, Hung-Chun Chen, Ming-Lung Yu, Shang-Jyh Hwang

**Affiliations:** 1 Division of Nephrology, Department of Internal Medicine, Kaohsiung Medical University Hospital, Kaohsiung Medical University, Kaohsiung, Taiwan; 2 Division of Gastroenterology, Department of Internal Medicine, Kaohsiung Medical University Hospital, Kaohsiung Medical University, Kaohsiung, Taiwan; 3 Division of Hepatobiliary, Department of Internal Medicine, Kaohsiung Medical University Hospital, Kaohsiung Medical University, Kaohsiung, Taiwan; 4 Faculty of Medicine, College of Medicine, Kaohsiung Medical University, Kaohsiung, Taiwan; 5 Faculty of Renal Care, College of Medicine, Kaohsiung Medical University, Kaohsiung, Taiwan; 6 Instrument Technology Research Center, National Applied Research Laboratories, Hsinchu, Taiwan; 7 Department of Internal Medicine, Kaohsiung Municipal Hsiao-Kang Hospital, Kaohsiung Medical University, Kaohsiung, Taiwan; IPO, Inst Port Oncology, Portugal

## Abstract

**Background:**

Chronic kidney disease (CKD) and hepatitis C virus (HCV) infection are closely linked and both increase patient mortality. The association of HCV and risk of developing end-stage renal disease (ESRD) has not been analyzed with competing risk model.

**Method:**

We enrolled a prospective cohort of 4,185 patients (mean age, 62 years; 41% female) registered in the CKD integrated care program at two affiliated hospitals of Kaohsiung Medical University in Taiwan between November 11, 2002 and May 31, 2009. With competing risk model, we analyzed the association of HCV infection, defined by seropositive of anti-HCV antibody, and hepatitis B virus (HBV) infection, defined by seropositive of HBV surface antigen, with the risk of entering ESRD.

**Results:**

The prevalence of HCV infection was 7.6% and it increased with the CKD stages (trend test, *P*<0.001), while the prevalence of HBV infection was 7.4% and no specific trend among CKD stages (tend test, *P* = 0.1). During the 9,101 person-year follow-up period, there were 446 death and 1,205 patients entering ESRD. After adjusting death as the competing risk, the estimated 5-year cumulative incidence rate of ESRD among patients with and without HCV infection were 52.6% and 38.4%, respectively (modified log-rank, *P*<0.001). Multivariable analysis showed that HCV infection, but not HBV infection, had higher risk of developing ESRD compared with cases without infection (HCV, HR: 1.32, 95% CI: 1.07–1.62; HBV, HR: 1.10, 95% CI: 0.89–1.35). Subgroup analyses showed consistent results.

**Conclusions:**

With death-adjusted competing risk analysis, HCV infection is associated with an increased risk of developing ESRD in CKD cohort.

## Introduction

Optimal care of chronic kidney disease (CKD) patients requires accurately estimating the risk of 2 competing clinical outcomes: end-stage renal disease (ESRD) and death [1]. Go and Wen have highlighted the increased mortality risk in reduced estimated glomerular filtration rate (eGFR) cases [2], [3]. The crude death–ESRD ratio by population studies have shown to be as high as 10–20 to one [4], [5]. When analyzing the risk of entering ESRD, death before ESRD actually prevents ESRD from occurring. Therefore, implementing competing risk model instead of traditional survival analysis should be considered [6]. Grams et al using the Modification of Diet in Renal Disease (MDRD) cohort demonstrated that method of analysis resulted in markedly different estimates. In CKD cases with vascular disease, the cumulated incidence by standard analysis that treating death as censored data showed that the 15-year incidence for ESRD was 66%, which was much higher than estimated with competing risk regression as 54%^.^ [1] With recently applied competing risk analysis, an old-aged CKD community cohort showed the rate (per 100 person-years) was 0.5 for ESRD and 6.8 for all-cause mortality [7]. Another study of CKD-outpatient-care-unit-based cohort showed the estimated rates (per 100 patient-years) of ESRD and death was 8.3 and 5.9 respectively [8]. The remarkably high ratio of death versus ESRD presented in previous studies and its impact on outcome analysis suggest the crucial role of competing analysis in ESRD outcome analysis of CKD population [1], [6].

Hepatitis C virus (HCV) infection, which affects 170 million people worldwide, is the leading cause of liver transplantation in developed countries and a risk factor for both mortality and kidney disease [9], [10]. HCV infection can cause cryoglobulinemia-induced membranoproliferative glomerulonephritis and other glomerulonephritis [11]. In 2012, the meta-analysis indicated that HCV infection was independently associated with proteinuria and not with reduced GFR. Heterogeneity between studies was mentioned [12]. Although the connection between HCV infection and kidney disease has proved in several studies[13]–[17], no study has applied the death adjusted competing risk model, and conventional survival analysis tends to overestimate risk when competing outcomes exists [1], [6]. In this study, we used a prospective CKD cohort with complete clinical data and definitive outcome categories to elucidate the association of HCV infection with risk of developing ESRD by using death adjusted competing risk analysis.

## Methods

### Study design and participants

The study was evaluated and approved by the Institutional Review Board at Kaohsiung Medical University Hospital (KMUH-IRB-980551). The review board waived the requirement for written informed consent because all identifying personal information was removed before analysis. The integrated CKD care program is a multidisciplinary care program that facilitate patient outcome in pre-ESRD preparation and early CKD education. All eligible consecutive cases that are followed up at our outpatient department and fulfilled the definition of CKD are invited to join this program. Comprehensive demographic and medical histories are recorded and prompt follow-ups along with the education program are carried out. It is a prospective CKD cohort with detailed clinical information and standardized care. Each patient's outcome would be recorded and validated. We selected patients from this cohort whose enrollment time was between the start of the program, November 11, 2002, and May 31, 2009.

During this period, 4,321 patients were enrolled in the integrated CKD care program at two affiliated hospitals, Kaohsiung Medical University Hospital and Kaohsiung Municipal Hsiao-Kang Hospital. After we excluded 28 cases that lacked follow-up serum creatinine data and 108 cases missing more than 20% of the variables, 4,185 patients remained in our study cohort.

### Measurements and definitions

All methods employed to assess the baseline characteristic of the CKD program cohort were described previously [18], [19]. In brief, demographic factors (age, sex, marital status, and education), health behaviors (herbal drugs use), comorbidity (diabetes mellitus, hypertension, and cardiovascular disease), physical examination findings (body mass index), and laboratory values (serum creatinine, albumin, blood hemoglobin, platelets, serum alanine aminotransferase, cholesterol, uric acid, glucose, and urine protein creatinine ratio) were ascertained at study entry. The laboratory data 3 months before and after enrollment in the CKD care system were averaged and analyzed [18], [19].

As in our previous study, HCV infection was defined by seropositive results for anti-HCV antibody, and hepatitis B virus (HBV) infection was defined by seropositive results for HBV surface antigen (HBsAg). Patients exhibiting no viral hepatitis data or seronegative results were defined as no HCV or HBV infection, respectively [20]. CKD staging was categorized according to the Kidney Disease Outcomes Quality Initiative (K/DOQI) definition, and eGFR was calculated using the 4-variable MDRD equation [21], [22]. Diagnoses of diabetes, hypertension, and cardiovascular disease were obtained from validated clinical records during enrollment [18], [19]. Mild and severe liver disease was categorized using International Classification of Diseases, Ninth Revision, Clinical Modification (ICD-9-CM) codes (mild liver disease: 70.3, 70.5, 70.7, 70.9, 570.0, 571.1, 571.3, 571.4, 571.8, 571.9, 573.x, v427, v0261.v0262; severe liver disease: 70.2, 70.4, 70.6, 571.2, 571.5, 571.6, 572.2–572.8, 456.0–456.2) [23].

The primary outcome of our study was ESRD. When a patient's renal failure progressed to ESRD requiring maintenance renal replacement therapy, catastrophic disease registry was performed. We obtained the ESRD data from our CKD program record and confirmed the event date by the National Health Bureau Catastrophic Disease Registry of renal replacement therapy. The competing risk outcome was death. Mortality was obtained from our CKD program record and the date of death was confirmed by the National Death Registry. Those patients free from events until the end of February, 2011 were considered as censored data. Time to event was calculated from the date of enrollment in the CKD integrated care program until ESRD, death, or the end of February, 2011.

### Statistical analyses

Baseline characteristics were summarized as mean ± standard deviation for continuous variables and frequencies and percentages for categorical variables. Differences among viral hepatitis groups were compared using chi-square test for categorical variables and *t*-test for continuous variables. Trend test was applied to compare the prevalence of viral hepatitis among CKD stages. Death before ESRD was considered a competing risk event. We calculated and compared the cumulative incidences of ESRD by modified Kaplan-Meier method and competing adjusted model, the Gray method [24].

To determine the independent risk factors for entering ESRD, multivariable analyses and stratified analyses, using hazard ratios, were conducted using modified Cox proportional hazard models in the presence of competing risk event after adjusting for age, sex, marital status, educational status, herb use, HBV infection, comorbidity (diabetes mellitus, hypertension, mild liver disease, sever liver disease, and cardiovascular disease), BMI, hemoglobin, platelets, albumin, alanine aminotransferase, cholesterol, uric acid, glucose, CKD stages and urine protein creatinine ratio [25]. All subgroup comparisons were preplanned to control for potential confounding factors reported in previous studies.

Data management and analyses were conducted using SAS 9.3 (SAS Institutes Inc., Cary, NC, USA). Figures were drawn using GraphPad Prism 5.0 (GraphPad Software Inc., San Diego, CA, USA) or R program with the ‘cmprsk’ package [26]. A *P* value below 0.05 was considered statistically significant.

## Results

### Participant's characteristics

**Figure 1:**
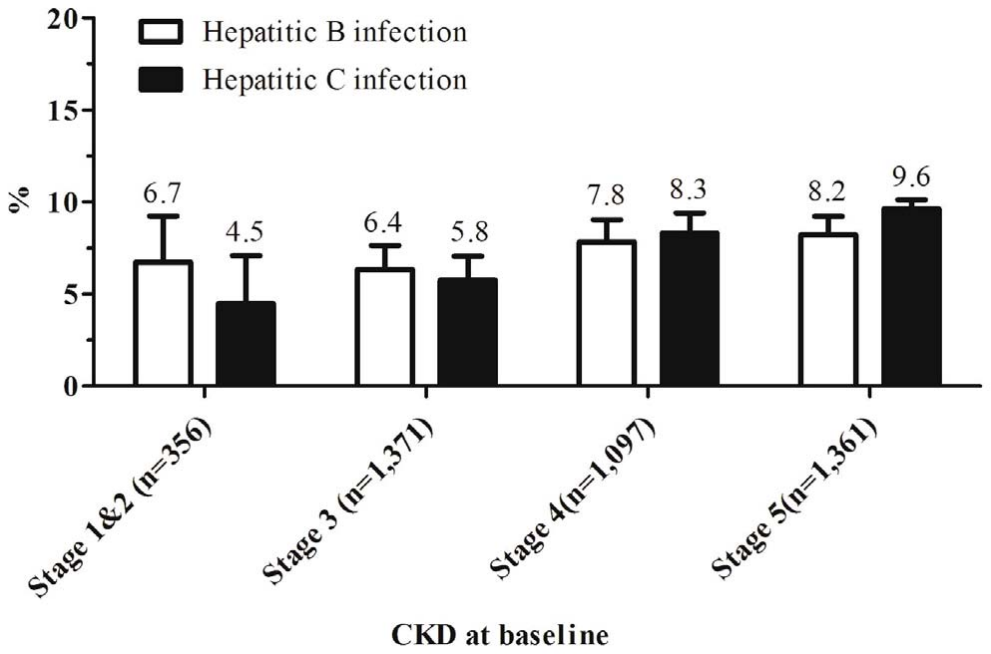
Prevalence of hepatitis B virus and hepatitis C virus infection at chronic kidney disease stages. Different prevalence between various stages of chronic kidney disease was analyzed by trend test. The *P* value was <0.001 in hepatitis C infected cases, and *P* = 0.1 in hepatitis B virus infected cases.

Our study consisted of 4,185 participants consecutively enrolled in the integrated CKD care program from November 11, 2002 to May 31, 2008, after excluding 28 cases with no follow-up serum creatinine and 108 cases with incomplete clinical variables. The mean age was 62.0±14.1 years, and 41% were female. The seroprevalence rate of HBsAg and anti-HCV were 7.4% and 7.6%, respectively (Table 1). Patients with HCV infection were more likely to be older, more female, lower education level, lower level of BMI, hemoglobin, platelets, albumin, cholesterol, but higher prevalence of comorbidities, and higher mean of alanine aminotransferase, uric acid, glucose, lower eGFR and more sever of proteinuria than patients without HCV infection (Table 1). Prevalence of HCV infection increased as the CKD stages advanced (trend test, *P*<0.001), and prevalence of HBV infection was nearly constant at various CKD stages (trend test, *P* = 0.1) **(** Figure 1 **)**.

**Table 1:** Baseline characteristics by status of HCV infection.

	Hepatitis C virus infection			
	Overall	Yes	None	P-value
Participants, *n*	4,185	317	3,868	
Age (y)	61.98±14.13	64.53±12.09	61.77±14.26	<0.001
Women	1,738 (41.5)	166 (52.4)	1,572 (40.6)	<0.001
Marital Status				
Yes	3,096 (75.4)	229 (73.2)	2,867 (75.6)	0.34
Educational status (y)				<0.001
0–6	2,092 (56.3)	209 (69.2)	1,883 (55.1)	
7–12	1,094 (29.4)	71 (23.5)	1,023 (29.9)	
> = 13	533 (14.3)	22 (7.3)	511 (15.0)	
Herb use				0.32
Yes	442 (11.2)	37 (13.0)	405 (11.1)	
Primary diseases				0.08
Chronic glomerular nephritis	1,531 (36.9)	103 (32.5)	1,428 (37.3)	
Diabetes mellitus	1,504 (36.2)	138 (43.5)	1,366 (35.6)	
Hypertension	456 (11.0)	29 (9.2)	427 (11.1)	
Tubulointerstitial nephritis	375 (9.0)	28 (8.8)	347 (9.1)	
Others	284 (6.8)	19 (6.0)	265 (6.9)	
Hepatitis B virus infection	309 (7.4)	25 (7.9)	284 (7.3)	0.72
Comorbidity				
Mild liver disease	576 (13.8)	119 (37.5)	457 (11.8)	<0.001
Sever liver disease	177 (4.5)	32 (10.1)	158 (4.1)	<0.001
Diabetes Mellitus	1,673 (40.0)	158 (49.8)	1,515 (39.2)	<0.001
Hypertension	2,553 (61.0)	224 (70.7)	2,329 (60.2)	<0.001
Cardiovascular disease	954 (22.8)	88 (22.4)	866 (22.4)	0.03
Laboratory data				
BMI (kg/m2)	24.75±4.03	24.14±3.94	24.8±4.03	0.006
Hemoglobin (g/dL)	11.15±2.47	10.35±2.22	11.22±2.48	<0.001
Platelets (x103/µL)	217.84±71.11	191.82±71.34	219.96±70.68	<0.001
Albumin (g/dL)	3.84±0.56	3.64±0.56	3.85±0.56	<0.001
ALT (U/L)	25.28±27.54	39.76±44.81	24.08±25.23	<0.001
Cholesterol (mg/dL)	197.43±55.53	182.72±53.47	198.63±55.53	<0.001
Uric acid (mg/dL)	7.8±1.99	7.93±2.07	7.79±1.99	0.24
Glucose (mg/dL)	115.89±44.64	118.91±50.31	115.64±44.13	0.26
eGFR (mL/min/1.73m2)	29.78±23.49	23.69±17.4	30.29±23.86	<0.001
Urine protein creatinine ratio (mg/mg)				<0.001
<1000	1,852 (46.7)	100 (32.7)	1,752 (47.9)	
1000–1999	866 (21.9)	69 (22.6)	797 (21.8)	
2000–2999	379 (9.6)	40 (13.1)	339 (9.3)	

Note: Data are expressed as number (percentage) for categorical variables and mean ± standard deviation for continuous variables. Statistical comparisons between viral hepatitis categories were performed using chi-square test for categorical variables and analysis of variance for continuous variables. eGFR was calculated using the 4-variable MDRD study equation.

Conversion factors for units: hemoglobin in g/dL to g/L, x10; serum albumin in g/dL to g/L, x10; serum cholesterol in mg/dL to mmol/L, x0.02586; serum uric acid in mg/dL to µmol/L, x59.48; serum creatinine in mg/dL to µmol/L, x88.4; serum glucose in mg/dL to mmol/L, x0.05551; eGFR in mL/min/1.73m2 to mL/s/1.73m2, x0.01667; Urine protein creatinine ratio in mg/mg to mg/mmol, x1.13; no conversion is necessary for platelet levels in 103/µL and 109/L.

Abbreviations: BMI, body mass index; ALT, alanine aminotransferase; eGFR, estimated glomerular filtration rate.

### Cumulative incidence of endpoints

**Figure 2:**
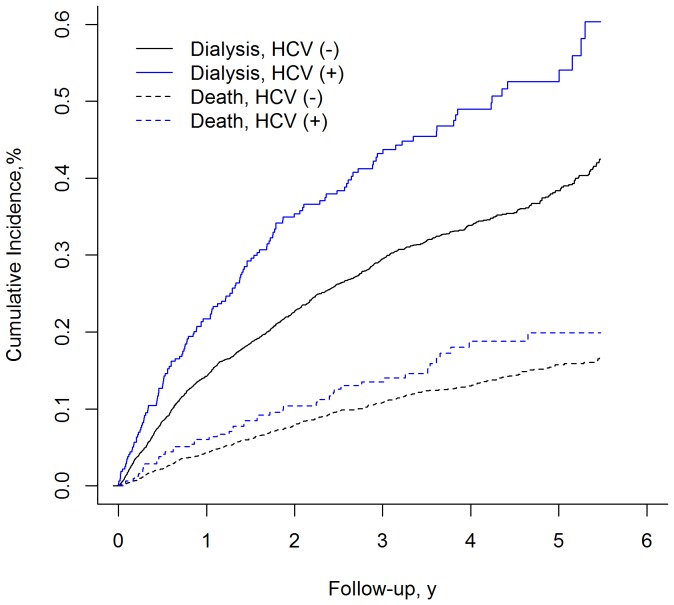
Cumulative incidence of end-stage renal disease adjusted competing for death plot showed HCV infection had higher cumulative rate of end-stage renal disease than cases without HCV infection (modified log-rank, *P*<0.001).

There were 446 death and 1,205 patients entered ESRD during a median ∼1.8 years, mean 2.2±1.6 years, and total 9,101 patient-years follow-up period. The rates (per 100 patient-years) of ESRD and death were 13.2% and 4.9%, respectively. The estimated cumulative incidence of ESRD was 49.0% using Kaplan-Meier method, and 39.6% using competing risk method. The 5-year cumulative incidence rate of ESRD adjusted by competing for death plot was significantly higher among HCV infection patients than those without HCV infection (52.6% vs. 38.4%, modified log-rank, *P*<0.001) (Figure 2).

### Risk of ESRD in patients with HCV infection

As detailed in table 2, the Cox proportional Hazards model with a competing risk framework showed that, with adjusting all possible covariates, patients with HCV infection but not HBV infection exhibited a higher risk of developing ESRD compared with patients without HCV or HBV infection (HCV: HR: 1.32, 95% CI: 1.07–1.62, *P* = 0.008; HBV: HR: 1.10, 95% CI: 0.89–1.35, *P* = 0.39). We also changed the CKD stages variable to initial eGFR in the multivariable adjusting model and had similar result (Data not shown). Other variable associated with increased risk for ESRD including younger age, male sex, mild liver disease, cardiovascular disease, lower hemoglobin, lower platelet, lower albumin, higher cholesterol, more advanced CKD stage, and more proteinuria (Table S1).

**Table 2:** Hazard ratios by status of viral hepatitis to end-stage renal disease adjusting competing risk of death.

	Age-adjusted		Age and multivariable-adjusted#	
	HR (95% CI)	p-value	HR (95% CI)	p-value
Hepatitis B infection				
None	1.00 [reference]		1.00 [reference]	
Yes	1.30 (1.08–1.57)	0.006	1.10 (0.89–1.35)	0.39
Hepatitis C infection				
None	1.00 [reference]		1.00 [reference]	
Yes	1.80 (1.50–2.14)	<0.001	1.32 (1.07–1.62)	0.008

Abbreviation: HR, hazard ratio; CI, confident interval.

Note: 3,646 patients presenting complete information were included in the multivariable analyses.

# Multivariables include sex, marital status, educational status, herb use, comorbidity (mild liver disease, diabetes mellitus, severe liver disease, hypertension, and cardiovascular disease), body mass index, hemoglobin, platelets, albumin, alanine aminotransferase, cholesterol, uric acid, glucose, CKD stages and urine protein creatinine ratio; HBV infection analysis adjusted status of HCV infection, and HCV infection analysis adjusted status of HBV infection.

### Subgroup analysis

**Figure 3:**
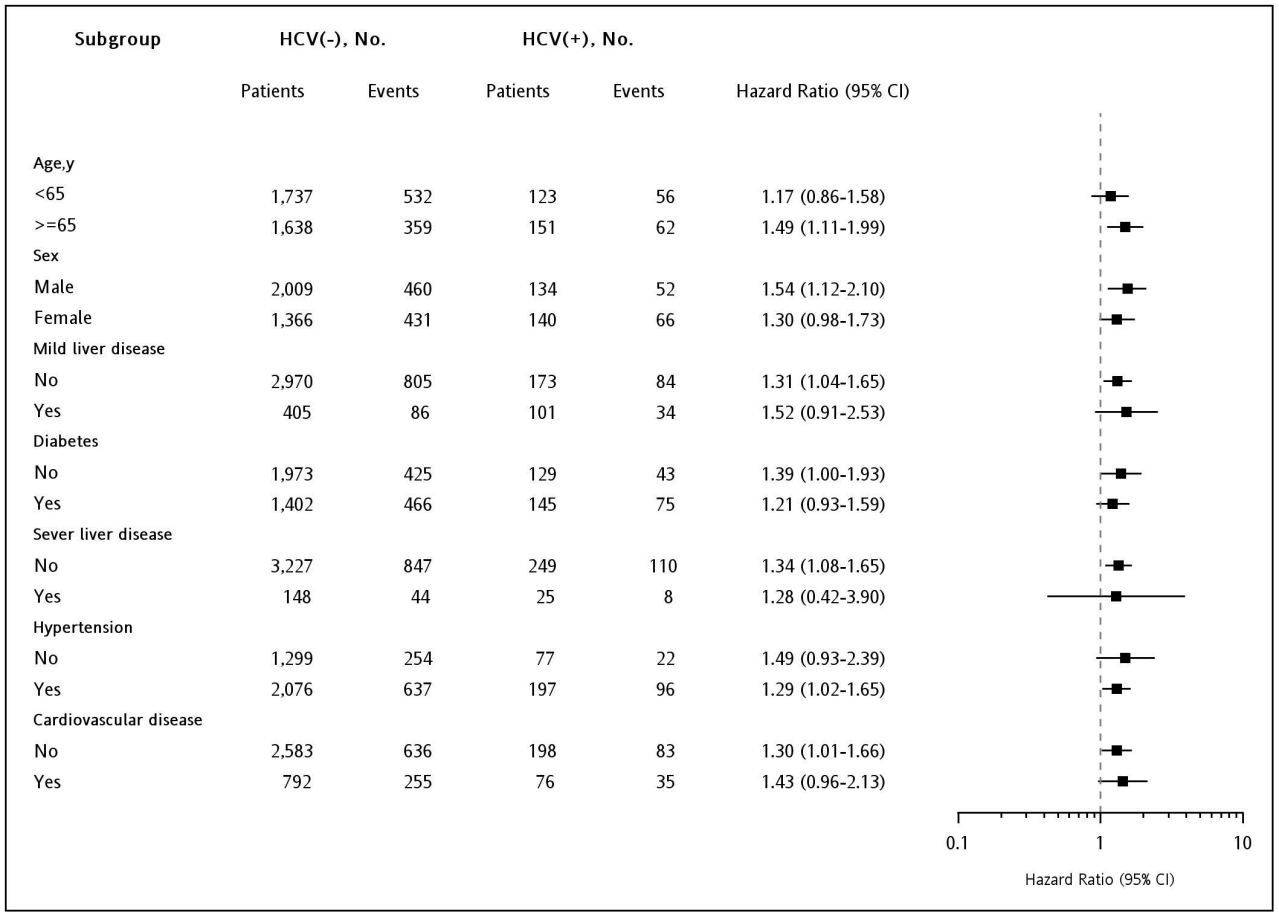
Multivariable stratified subgroup analyses for the association of hepatitis C virus infection with the risk of entering end-stage renal disease.

All subgroup comparisons were preplanned to control for potential confounding factors, such as age, sex, diabetes, hypertension, cardiovascular disease, and liver disease, reported in previous studies. The results of all subgroup analyses presented similar and consistent findings that HCV infection increased the risk of entering ESRD (Figure 3).

## Discussion

This study first applied the death adjusted competing risk model to analyze the association between HCV infection and the risk of developing ESRD in CKD cohort. The results indicated that HCV infection presented a high cumulative rate of ESRD and was an independent risk factor for developing ESRD. Further stratified subgroup analysis including age, sex, diabetes, hypertension, cardiovascular disease, and liver disease presented consistent results. Prevalence of HCV infection was also increased in advanced CKD stages.

All 4 previous cohort studies favored that HCV infection associated with increased risk of developing ESRD [14]–[17]. However, CKD and HCV patients have a higher death risk compared with general population [2], [3], [27]. The crude ratio of death to ESRD was approximately 3.7 to 10 in our study. This ratio was lower than data from community studies and more close to data from hospital-cared CKD cohorts [4], [5], [7], [8]. Reasons for the lower ratio of death to ESRD might be due to the CKD care cohort used, as well as a relatively shorter follow-up period. However, in this study, HCV infection group having lower eGFR and higher prevalence in advanced CKD stages was compatible to our large-scale community observation (Table 1, Figure 1) [20]. It have been studied at the existence of competing risk, conventional survival analysis overestimates the risk of event of interest [1], [6]. The overestimation was observed in this study with overall cumulative incidence of ESRD as 49.0% according to the standard survival analysis (Kaplan-Meier method, censoring at the competing event) and decreased to 39.6% based on competing risk method. In the competing risk framework, the 5-year cumulative incidence rate of ESRD in HCV infection cases was 52.6%, which was higher than cases without HCV infection as 38.4% (modified log-rank, *P*<0.001) (Figure 2). Base on the results of multivariable analyses adjusting competing risk of death and confounders, we further confirmed and strengthened previous researches that HCV infection independently and considerably increased risk for ESRD.

In our multivariable analysis, older age was associated with the lower risk of ESRD (Table S1). Age-dependent effect was acknowledged by Tsui's report, which indicated that HCV infection increased the risk of ESRD in the younger age group, but not in the older than 70 years age group [15]. Age-stratified analysis in Su's study determined that the association between HCV and ESRD presented in all age groups, but the size of effect seemed weaker in the older age group [17]. This finding might be related to the close linkage between older age and death. Conway et al used an older CKD cohort and determined that the risk of surviving to requiring dialysis decreased as age increased, and two to one cases died before entering ESRD [28]. A community-based cohort exhibited an even higher ratio of death versus ESRD in the older age group [7]. Using competing risk analysis, Nicola et al showed younger age predicted ESRD, whereas older age predicted death [8]. All these findings emphasized and supported the importance of considering death as a competing outcome when analyzing risk for ESRD. Additionally, no gender effect was observed. Sex-stratified analysis in Su's study indicated that women and men exhibited a similar risk for HCV infection to increase ESRD [17]. We also stratified crucial comorbidities for clarifying the association between HCV infection and ESRD, such as diabetes [14], [15], hypertension, and cardiovascular disease [1], [2]. Satapathy et al reported that a high baseline HCV viral load was an independent predictor of CKD, and HCV infection presented shorter renal survival [16]. The viral load data were unavailable for most of our cases; thus, we performed liver disease-stratification analysis. All of these subgroup analyses presented consistent results that HCV infection increased risk for entering ESRD (Figure 3).

The linkage of HCV infection and kidney disease is complex. HCV infection can induce cryoglobulinemia-associated membranoproliferative glomerulonephritis, and is associated with other glomerular manifestations in both native and transplanted kidneys [11]. However, direct evidence for HCV causing renal injury and renal failure remains limited, possibly because of lack of experimental animal models [29]–[31]. In addition, increased insulin resistance and risk of diabetes and metabolic syndrome in HCV infected patients could also be a contributor to renal injury and renal progression [32], [33]. Currently, more studies to demonstrate and elucidate the causal relationship between HCV infection and kidney disease are necessary. Although Fabrizi's meta-analysis determined that HCV infection was associated with proteinuria and not with reduced GFR in the general population, substantial heterogeneity between studies was observed [12]. Proteinuria is a well-known CKD progression factor and is also a marker of kidney injury. Their result did not completely exclude the possibility of HCV related to CKD. More and more evidence from current studies supports the relationship. Chen indicated that HCV was a causal risk factor for incidence of CKD through national claim data [13]. Satapathy et al, using a gastroenterology outpatient cohort, determined that HCV infection was an independent positive predictor, whereas history of interferon treatment was a negative predictor of CKD [16]. The negative predictor of CKD in interferon treatment and beneficial result in treating cryoglobulinemic-glomerulonephritis suggested the potential requirement for reconsidering the treatment strategy for HCV patients [11], [16], [34]. Although using HCV treatment to achieve a sustained viral response is difficult in advanced renal failure because of complications, it is a requirement for a successful and uneventful kidney transplantation [10]. The timing for HCV treatment in an earlier disease process may improve the clinical outcome and prevent organ failure. Hsu et al recently reported a promising result that antiviral treatment improved renal outcomes in a diabetes cohort [35]. Additional clinical trials on HCV treatment of the CKD cohort and studies on the pathophysiological mechanism are necessary.

Our study has the advantages compared to studies based on claim datasets. First, the accuracy of HCV diagnoses in our study through serology test is expected to be better than definitions by disease coding from claim data. Second, the early check of Anti-HCV in the phase of enrollment into CKD care program balances the time-related bias, and overcomes the drawback of diagnosis of HCV infection too close to date of dialysis initiation. Finally, plenty of clinical data were collected for adjustment through competing risk approach to reduce the potential confounding bias provides a more correct estimation of ESRD risk. However, several limitations to our study still need to be mentioned. First, we defined HCV infection as seropositive results for anti-HCV antibody rather than the presence of serum HCV RNA. Approximately 25% of anti-HCV seropositive patients might spontaneously clear the virus [36], [37]. However, HCV RNA data were not available for most of our study cohort. We could not evaluate the impact of HCV viremia and baseline viral load on the progression of CKD to ESRD. Thus, we attempted to perform subgroup analysis stratified with mild and severe liver disease, which showed consistent results. Second, the status of human immunodeficiency virus (HIV) was unavailable. Co-infection of HIV and HCV is an emerging public problem worldwide, particularly in injection drug users [38]. However, we believe that rare HIV cases (0.01%) in ESRD population in Taiwan diminished the impact of HIV status in our study result.

In conclusion, our study first applied death adjusted competing risk analysis, confirmed that HCV infection associated with increased risk of CKD patients entering ESRD. This substantial clinical significance suggests that future investigations on HCV treatment and renal outcome are needed to elucidate better care for CKD population.

## Supporting Information

Table S1 **Hazard ratios for end-stage renal disease adjusting competing risk of death by multivariable analysis.**
(DOCX)
