# Supplementary material for: Hepatitis C Virus Infection Increases Risk of Developing End-Stage Renal Disease Using Competing Risk Analysis
Source: PLoS One. 2014 Jun 27;9(6):e100790. doi: 10.1371/journal.pone.0100790 (PMC4074067; doi:10.1371/journal.pone.0100790)
Supplement: Table S1 — Hazard ratios for end-stage renal disease adjusting competing risk of death by multivariable analysis. (DOCX) [file pone.0100790.s001.docx]

Table S1. Hazard ratios for end-stage renal disease adjusting competing risk of death by multivariable analysis

| Variable | Adjusted-HR | (95% CI) | P-value |
| --- | --- | --- | --- |
| Hepatitis C virus infection | | | |
| No | 1.00 | [reference ] |  |
| Yes | 1.32 | (1.07-1.62) | 0.008 |
| Age, per year increase | 0.98 | (0.98-0.99) | <0.001 |
| Sex | | | |
| Male | 1.00 | [reference ] |  |
| Female | 0.52 | (0.45-0.60) | <0.001 |
| Educational level | | | |
| 0-6 | 1.00 | [reference ] |  |
| 7-12 | 0.89 | (0.76-1.04) | 0.1 |
| >=13 | 0.84 | (0.67-1.05) | 0.1 |
| Primary diseases | | | |
| Chronic glomerular nephritis | 1.00 | [reference ] |  |
| Diabetes mellitus | 1.14 | (0.94-1.39) | 0.2 |
| Hypertension | 1.01 | (0.78-1.31) | 0.9 |
| Tubulointerstitial nephritis | 0.83 | (0.62-1.11) | 0.2 |
| Others | 0.99 | (0.74-1.33) | 0.9 |
| Mild liver disease | | | |
| No | 1.00 | [reference ] |  |
| Yes | 0.75 | (0.61-0.93) | 0.008 |
| Sever liver disease | | | |
| No | 1.00 | [reference ] |  |
| Yes | 1.03 | (0.77-1.38) | 0.8 |
| Herb use | | | |
| No | 1.00 | [reference ] |  |
| Yes | 1.07 | (0.91-1.25) | 0.4 |
| Diabetes Mellitus | | | |
| No | 1.00 | [reference ] |  |
| Yes | 1.10 | (0.92-1.32) | 0.3 |
| Hypertension | | | |
| No | 1.00 | [reference ] |  |
| Yes | 1.09 | (0.93-1.27) | 0.3 |
| Cardiovascular disease | | | |
| No | 1.00 | [reference ] |  |
| Yes | 1.25 | (1.08-1.46) | 0.004 |
| Hepatitis B virus infection | | | |
| No | 1.00 | [reference ] |  |
| Yes | 1.10 | (0.89-1.35) | 0.4 |
| Hemoglobin, per 1 g/dL increase | 0.78 | (0.74-0.82) | <0.001 |
| Platelets, per 1 103/μL increase | 0.998 | (0.997-0.999) | <0.001 |
| Albumin, per 1 g/dL increase | 0.54 | (0.47-0.62) | <0.001 |
| ALT, per 1 U/L increase | 0.998 | (0.995-1.001) | 0.2 |
| Glucose, per 1 mg/dL increase | 0.999 | (0.998-1.001) | 0.3 |
| Cholesterol, per 1 mg/dL increase | 1.003 | (1.002-1.005) | <0.001 |
| Uric acid, per mg/dL increase | 1.02 | (0.99-1.05) | 0.1 |
| BMI, per 1 kg/m2 increase | 0.99 | (0.98-1.01) | 0.3 |
| CKD stage | | | |
| Stage 1&2 | 1.00 | [reference ] |  |
| Stage 3a | 1.71 | (0.19-15.35) | 0.6 |
| Stage 3b | 4.75 | (0.65-34.71) | 0.1 |
| Stage 4 | 23.36 | (3.23-169.10) | 0.002 |
| Stage 5 | 94.38 | (13.03-683.88) | <0.001 |
| Urine protein creatinine ratio (mg/mg) | | | |
| <1000 | 1.00 | [reference ] |  |
| 1000-1999 | 1.51 | (1.24-1.85) | <0.001 |
| 2000-2999 | 2.42 | (1.91-3.06) | <0.001 |
| >=3000 | 3.00 | (2.43-3.70) | <0.001 |

Abbreviation: HR, hazard ratio; CI, confident interval
